# L-Phenylalanine Transport in *Saccharomyces cerevisiae*: Participation of *GAP1, BAP2,* and *AGP1*

**DOI:** 10.1155/2014/283962

**Published:** 2014-02-20

**Authors:** Daniel A. Sáenz, Mónica S. Chianelli, Carlos A. Stella

**Affiliations:** Departamento de Bioquímica Humana, Facultad de Medicina, Paraguay 2155, 1121 Buenos Aires, Argentina

## Abstract

We focused on the participation of GAP1, BAP2, and AGP1 in L-phenylalanine transport in yeast. In order to study the physiological functions of GAP1, BAP2, and AGP1 in L-phenylalanine transport, we examined the kinetics, substrate specificity, and regulation of these systems, employing isogenic haploid strains with the respective genes disrupted individually and in combination. During the characterization of phenylalanine transport, we noted important regulatory phenomena associated with these systems. Our results show that Agp1p is the major transporter of the phenylalanine in a gap1 strain growing in synthetic media with leucine present as an inducer. In a wild type strain grown in the presence of leucine, when ammonium ion was the nitrogen source, Bap2p is the principal phenylalanine carrier.

## 1. Introduction

Amino acid transport across the *Saccharomyces cerevisiae* plasma membrane is an active and highly regulated process mediated by transmembrane proteins called permeases.Based on their amino acid sequences, these permeases can be grouped into an amino acid permease family (AAP) that includes 18 members of proteins [1–4].

Amino acid permeases display different specificities and capacities that allow extracellular amino acids to be used either as a general nitrogen source or as building blocks in protein synthesis.

The high-capacity permeases Agp1p, Gap1p, and Put4p are expressed under conditions where amino acids are needed as a source of nitrogen to support growth and are preferentially active in cells growing on poor nitrogen sources, such as allantoin, urea, or proline. When a good nitrogen source such as glutamine, asparagine, or ammonium ions is present, utilization of the poorer nitrogen sources is greatly diminished. This general phenomenon is the result of at least two different regulatory mechanisms: nitrogen catabolic repression (NCR), which affects the synthesis of these permeases [5–8], and nitrogen catabolic inactivation (NCI), which downregulates the activity of several permeases that import these poor nitrogen sources.

Although the sequences of the 18 AAP genes are known and the corresponding amino acid sequences deduced, the kinetic properties of many of the more specific permeases have not been described.

It has been shown that L-phenylalanine is required for *S. cerevisiae* optimal growth on synthetic media [9, 10]. However, only limited information concerning the specific permeases involved in the L-phenylalanine transport has been published. For years, it was believed that phenylalanine is transported primarily or exclusively by the general amino acid permease [11]. Although several investigators have observed effects of certain mutations on the transport of phenylalanine [12–14], no detailed study of phenylalanine transport has been made [14], nor has a phenylalanine-specific permease been defined [12]. In a strain disrupted in *GAP1*, additional disruption of *AGP1* greatly reduced growth of yeast on medium containing 1.0 mM phenylalanine as the sole nitrogen source [13]. Although Regenberg et al. [14] observed phenylalanine uptake in a *gap1ssy1* strain overexpressing *BAP2*, Grauslund et al. [15] observed no change in aromatic amino acid uptake under different conditions.

In the present study, we focused on the participation of *GAP1* [16], *BAP2 *[15, 17], and *AGP1* [13, 18] in L-phenylalanine transport. The Bap2p and Agp1p permeases belong to a subset of more related AAPs (Cluster I) [3] that also includes Gnp1p [19], Bap3p [20–22], Tat1p, and Tat2p [23, 24]. The expression of genes encoding the Cluster I permeases is induced by the presence of amino acid in the growth media and requires *SSY1*, *PTR3*, and *SSY5* gene-products [4, 12, 13, 21, 25]. These genes are essential components of a signalling pathway (SPS pathway) that activates transcription of multiple permease genes.

Schreve et. al [18] identified Agp1p as an asparagine and glutamine permease. It was further suggested that Agp1p can act as a low-affinity, broad-substrate-range permease that is involved in the uptake of amino acids for catabolism. Subsequently, Iraqui et al. [13] showed that Agp1p could transport most neutral amino acids, including phenylalanine with *K*_*T*_ = 0.6 mM. Regenberg et al. [14] employed overexpression and deletion to show that *BAP2*, *AGP1*, *BAP3*, and *TAT2* could be involved in phenylalanine uptake. All of these permeases have previously been identified as transporters for other amino acids.

In order to study the physiological functions of *GAP1*, *BAP2*, and *AGP1* in L-phenylalanine transport, we examined the kinetics, substrate specificity, and regulation of these systems, employing isogenic haploid strains with the respective genes disrupted individually and in combination. During the characterization of phenylalanine transport we noted important regulatory phenomena associated with these systems. Our results show that Agp1p is the major transporter of the phenylalanine in a gap1 strain growing in MA or MP media with leucine present as an inducer. In a wild type strain grown in the presence of leucine in MA medium, Bap2p is the principal phenylalanine carrier.

## 2. Materials and Methods

### 2.1. Strains and Media

The *Saccharomyces cerevisiae* strains used in this study are all isogenic with the “wild type” parental strain Y294, *MATαura3-52 leu 2-3,112 his3*-Δ*1 trp1-289* [17] except for the mutations mentioned; Y294Δbap2 with *bap2::URA3*; Y294Δgap1 with *gap1::LEU2*; Y294Δbap2Δgap1 with *bap2::URA3* and *gap1::LEU2*; [17]; Y294 Δagp1 with *agp1::URA3*, and Y294Δagp1Δgap1 with *agp1::HIS3 *and* gap1::LEU2*. The parental strain and all the disruption strains were the generous gift of Garrett.

For general maintenance of yeast cultures, YPD medium (1% yeast extract (Difco), 2% peptone (Difco), and 2% D-glucose) was used. Minimal ammonium (MA) medium contained per liter: 1.7 g Yeast Nitrogen Base without ammonium sulfate and amino acids (Difco), 5 g ammonium sulfate, and 2 g D-glucose. MP medium, and MG or MAA medium were similar to MA except that 1 g of L-proline, 10 mM of L-glutamine, or 1 mM of an L-amino acid was used as sole nitrogen source. When solid media were required, 2% (w/v) Bacto Agar (Difco) was included. All minimal media contained the following supplements unless indicated otherwise: uracil (20 mg/L), L-histidine (20 mg/L), L-tryptophan (20 mg/L), and L-leucine (30 mg/L = 0.23 mM). Assays for resistance to toxic analogues were carried out on plates of MA-leu (MA medium without L-leucine). Concentrations of analogues used were as follows: 15 *μ*g/mL *β*(2-thienyl)-DL-alanine (Sigma Chemical Co.); 9 *μ*g/mL 5,5,5-trifluoro-DL-leucine (Fairfield Chemical Co.).

### 2.2. Amino Acid Uptake

Two hundred microliters from an overnight culture was used to inoculate 50 mL of culture medium as indicated in each case and grown to a cell density corresponding to an OD_570_ of 1.0 to 2.0 at 30°C. The cells were harvested, washed twice with 50 mL of distilled water, and resuspended in distilled water at OD_570_ of 4.5. The uptake measurements were performed as described by Chianelli et al. [26]. The reaction mixture containing 0.5 mg cell dry weight mL^−1^ in 20 mM potassium phthalate buffer (pH 4.5) was incubated at 30°C with shaking. The reaction was initiated by the addition of 0.1–0.3 *μ*ci of L-[^14^C]-phenylalanine at a final concentration of 20 *μ*M or 1.0 mM. Samples of 0.2 mL were withdrawn at 0 (7 sec), 2, and 4 min in order to determine the initial uptake rate. Uptake saturation kinetics were measured at phenylalanine concentrations between 0.005 and 2.0 mM (specific activity between 0.25 and 10.00 *μ*ci/*μ*mol). The initial rates of uptake were estimated from the initial parts of the curves by linear regression and are expressed as *μ*mol g^−1^ cell dry weight min^−1^. All uptake measurements are the average of 2-3 separate experiments. Data were fitted to Michaelis-Menten and Lineweaver-Burk plots using the Solver program of Microsoft Excel 5.0 (Microsoft Corp., Bellevue, WA). The phenylalanine uptake data of Y294 Δbap2 and Y294 were fitted assuming two and three transport systems, respectively. The Solver program was used to simultaneously fit the Y294 with the parameters obtained from the Y294Δbap2 data for the two permeases and by adjusting the Bap2 parameters to obtain the best fit. All radiolabelled amino acids used were obtained from New England Nuclear. L-amino acids were purchased from Sigma Chemical Co.

## 3. Results

### 3.1. Disruption of *BAP2* in a *gap1* Strain Confers Resistance to Toxic Analogues of Leucine and Phenylalanine

An initial indication that Bap2p could transport phenylalanine was obtained by demonstrating that on MA plates a *bap2 gap1* strain was resistant to the phenylalanine analogue *β*-(2-thienyl) DL-alanine and to the leucine analogue DL-trifluoroleucine [26] (data not shown) when compared to an isogenic *gap1* strain. This result confirms the finding that overexpression of *BAP2* in *gap1ssy1* cells led to substantial phenylalanine uptake [14].

### 3.2. Disruption of *AGP1* in a *gap1* Strain Reduces Growth on Branched-Chain and Aromatic Amino Acids

We compared the growth of the *gap1*, *gap1bap2*, and *gap1agp1* strains on a low concentration (1.0 mM) of several individual amino acids: leucine, isoleucine, phenylalanine, tryptophan, tyrosine, and threonine, each used as the sole nitrogen source. The *gap1bap2* mutant displayed no clear growth defect on any of the amino acids. Interestingly, this growth test failed to show any contribution of Bap2p, defined as the major branched-chain amino acid permease in the utilization of leucine or isoleucine as the sole nitrogen source. However, disruption of the *AGP1* gene in the *gap1* strain reduced the growth on low concentrations of leucine, isoleucine, phenylalanine, tryptophan, tyrosine, and threonine (data not shown).

Agp1p has been reported to be a permease for most neutral amino acids [13, 14], a finding which is confirmed by the present results. We must note that the concentration of the amino acid used as the major nitrogen source is five times higher than the concentration of the amino acid used to compensate for the strain auxotrophies.

### 3.3. Activation of Phenylalanine Transport by Extracellular Leucine

Two important factors that influence phenylalanine transport activity in yeast are the presence of amino acids in the growth medium and the existence of a signalling pathway (SPS or ssy1-dependent pathway). This pathway senses external amino acids and transmits a transcriptional activation impulse to multiple AAP genes, including *BAP2* and *AGP1* [12, 13, 21].

Because the deletion mutants used in this study contain different combinations of auxotrophic markers for histidine, leucine, and tryptophan, all growth media contained these three amino acids, together with uracil, unless indicated otherwise. The phenylalanine uptake assay is performed in washed cells suspended in FHK buffer. Thus, the amino acids necessary to compensate for auxotrophies are absent and do not exert a competitive effect on the phenylalanine uptake. The concentration of leucine used was 0.23 mM (30 mg/mL). This leucine concentration was used by Didion et al. [21] to induce transcription of the amino acid permease genes *BAP2*, *BAP3*, and *TAT1*. Forsberg et al. [4, 25] used 0.15 mM leucine to activate transcription of several additional permease genes, including *AGP1*, via the SPS signalling pathway. As shown in Table 1, addition of leucine caused an increase of 64% in the initial velocity values in phenylalanine uptake in the Y294Δgap1 strain. Because the medium already contains 0.10 mM tryptophan, a good signalling activator [4, 13, 21, 25], addition of 0.23 mM leucine caused only a small additional effect.

### 3.4. Disruption of *BAP2* or *AGP1* Leads to a Reduced Uptake of L-Phenylalanine

**Figure 1:**
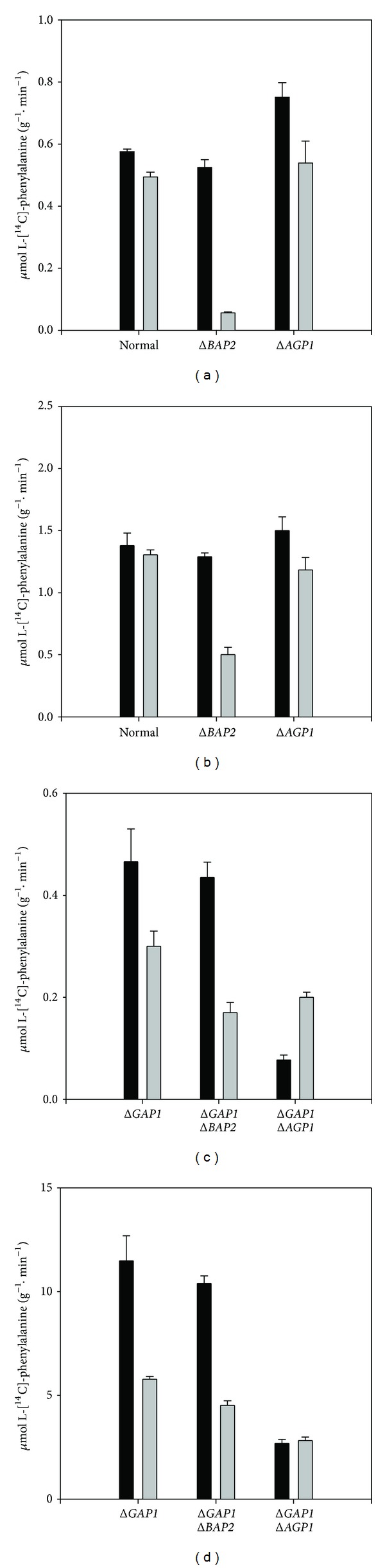
Phenylalanine uptake into Y294 wild type and mutant yeast strains inactivated in *GAP1*, *BAP2*, and *AGP1*. The strains were grown in MP (black bars) or MA (shaded bars) media containing leucine, histidine, tryptophan, and uracil. The L-[^14^C]-phenylalanine uptake was assayed as described in Section 2. Phenylalanine uptake was determined at an amino acid concentration of 20 *μ*M ((a) and (c)); phenylalanine uptake was determined at amino acid concentration of 1.0 mM ((b) and (d)).

To investigate directly the involvement of Gap1p, Bap2p, and Agp1p in L-phenylalanine transport in *S. cerevisiae*, we measured phenylalanine uptake into isogenic yeast strains bearing different mutations under various experimental conditions. The uptake rates were determined in cells grown in both MP and MA media and at two different substrate concentrations (Figure 1). At low substrate concentrations, uptake occurs by high-affinity permeases, whereas at high substrate concentrations, amino acid uptake occurs predominantly through low-affinity permeases.

For a number of L-amino acids, namely, glycine, alanine, phenylalanine, tryptophan, and tyrosine [11, 27], Gap1p has been claimed to be the principal transporter in *S. cerevisiae* cells. The data in Figure 1 indicate that in the absence of general amino acid permease activity, transport of phenylalanine may involve Bap2p, Agp1p, and at least one additional transport system. The relative contribution of these alternative systems depends both on the type of nitrogen source and the concentration of phenylalanine available.

As seen from Figures 1(a) and 1(b), in Y294 and Y294Δagp1 strains a minor decrease in phenylalanine uptake is evident in MA medium, as compared to MP medium. This indicates that Gap1p inactivation by ammonium ion has very limited or no effect on phenylalanine uptake. In contrast, a very important difference in the uptake rate was found when phenylalanine uptakes by Y294Δbap2 yeast cells grown in the two different media were compared. As previously reported, the general amino acid permease, Gap1p, is strongly repressed and inactivated in MA medium [6, 16]. Inactivation of Gap1p by the ammonium ion in MA medium is confirmed by the results shown in Table 2.

Uptake of L-citrulline, which is transported only by Gap1p, [16] is negligible in wild type and in both the Y294Δbap2 and Y294Δagp1 mutants, when cells were grown in MA medium. In MP medium, however, all three strains exhibit Gap1p activity, as measured by L-citrulline transport. Whereas the phenylalanine uptake by the three strains grown in MP may be attributed largely to uptake by Gap1p, the uptake by cells grown in MA is catalyzed mainly by Bap2p. Disruption of *BAP2 *reduces high-affinity phenylalanine transport to about 10% of the wild type level (Figure 1(a)). At high substrate concentration, low-affinity phenylalanine transport in MA-grown cells also depends upon functional *BAP2*, but to a lesser extent (Figure 1(b)). Deletion of *BAP2* reduces high-affinity phenylalanine transport only 61%. The residual uptake is not due to Agp1p, since deletion of *AGP1* has almost no negative, effect irrespective of phenylalanine concentration (Figures 1(a) and 1(b)).

Deletion of *GAP1* causes a minor (20%) decrease in high-affinity phenylalanine uptake in MP-grown cells (Figures 1(a) and 1(c)), but an eightfold increase in low-affinity phenylalanine transport was relative to wild type (Figures 1(b) and 1(d)). For MA-grown cells the increase was 4.4-fold. Because the additional deletion of *BAP2* (Figure 1(d)) causes only minor changes, it is unlikely that *BAP2* is responsible for this high uptake. In contrast, deletion of *AGP1* in a *gap1* strain causes dramatic decreases in both high- and low-affinity phenylalanine uptake in MP-grown cells (Figures 1(c) and 1(d)), indicating that Agp1p is acting as the primary phenylalanine carrier in *gap1* strains.

In MA-grown cells, Bap2p and Agp1p both appear to contribute to both high- and low-affinity phenylalanine uptake (Figures 1(c) and 1(d)). In contrast, when only *AGP1* was disrupted, phenylalanine transport was not notably altered in cells grown in either MA or MP medium (Figures 1(a) and 1(b)). Clearly, *GAP1* disruption activates *AGP1* expression, whereas inactivation/repression of *GAP1* does not. (Compare Figures 1(c) and 1(d).)

### 3.5. Specificity of L-Phenylalanine Transport Systems

It was of interest to determine the specificity of various amino acid permeases for phenylalanine in normal and mutant strains by measuring phenylalanine uptake in the presence of various amino acids and analogues at tenfold higher concentrations.

The results presented in Table 3 were obtained using MP-grown cells in which Gap1p is active in single-deletion strains as well as in wild type. As expected, all amino acids tested, including citrulline, exhibited some competition with phenylalanine. Although the pattern of inhibition was nearly the same in wild type and in the *agp1* strain, the *bap2* strain exhibited substantially greater inhibition by valine, tyrosine, tryptophan, asparagine and, most notably, by citrulline, and less by DL-trifluoroleucine. These results suggest that elimination of Bap2p activity, but not of Agp1p activity, causes the cell to utilize Gap1p to a greater degree. In other words, even in wild type MP-grown cells, Bap2p makes a significant contribution to the high-affinity phenylalanine uptake.

Additional evidence that Bap2p contributes significantly to high-affinity phenylalanine uptake in MA-grown cells is given in Table 4. Deletion of *BAP2* greatly reduces competition by leucine and almost eliminates sensitivity to DL-trifluoroleucine. Nevertheless, the residual phenylalanine transport exhibits some sensitivity to the phenylalanine analogue *β*-(2-thenyl) alanine. The results shown in Tables 3 and 4 clearly indicate that (1) the general amino acid permease is the main phenylalanine transporter in the Y294Δbap2 strain grown in MP medium; (2) in wild type and Y294Δagp1, besides Gap1p, another permease, presumably Bap2p, contributes to the transport of phenylalanine in MP medium; (3) in cells grown in MA medium, phenylalanine transport by Bap2p is inhibited specifically by trifluoroleucine (48–57%).

Under the same experimental conditions the inhibition profiles of phenylalanine uptake are quite different in the *gap1* strains (Table 5) as compared to the *GAP1* strains (Tables 3 and 4).

The results shown in Table 5 provide further evidence that Agp1p is an important transporter in MP-grown cells lacking *GAP1*. Leucine, tryptophan, and particularly asparagine compete with phenylalanine for transport. Moreover, absence of *BAP2* has no effect on the inhibition pattern with these three amino acids. However, when cells are grown in MA, two major differences can be seen. (1) Whereas in Y294Δgap1 and Y294Δbap2Δgap1 phenylalanine uptake is inhibited by asparagine, this inhibition is lost in strain Y294Δagp1Δgap1. This indicates that phenylalanine transport in MA-grown cells involves Agp1p. (2) The participation of Bap2p in high-affinity phenylalanine transport in both Y294 Δgap1 and Y294 Δagp1Δgap1 strains is supported by the strong inhibition by leucine and DL-trifluoroleucine. When *BAP2* is deleted, the trifluoroleucine inhibition almost disappears, and there is a significant decrease in leucine inhibition. The inhibition of phenylalanine uptake by tryptophan in MA-grown cells disrupted in both *GAP1* and *BAP2,* or *GAP1* and *AGP1*, suggests the possible participation of a *TAT* gene-product. This might also account for the resistance of the Y294Δbap2Δgap1 strain to *β*-(2-thienyl) alanine. This might also account for the sensitivity of the Y294 and Y294Δagp1Δgap1 strains to *β*-(2-thienyl) alanine.

It can be seen in Table 5 that in cells grown in MA medium, the most significant decreases in the inhibition of the phenylalanine uptake were observed with trifluoroleucine in the Y294Δbap2Δgap1 strain and with asparagine in the Y294Δagp1Δgap1 strain.

### 3.6. Kinetic Analysis of L-Phenylalanine Transport by Bap2p and Agp1p

In order to study in more detail the involvement of Gap1p, Bap2p, and Agp1p in phenylalanine transport we examined the kinetics of phenylalanine uptake by cells of wild type strain Y294 and mutant yeast strains grown in MP and MA media (Table 6). L-[^14^C]-phenylalanine uptake was determined at a range of phenylalanine concentrations and results were plotted as Michaelis-Menten or as Eadie-Hofstee graphs (data not shown).

In cells grown in MP medium, phenylalanine transport is clearly biphasic. The Eadie-Hofstee plots indicate that phenylalanine uptake is mediated by a component of high affinity, presumably Gap1p. The second component either is nonsaturable, or has very low affinity. Computer analysis of these uptake data provided the kinetic parameters given in Table 6. In cells grown in MP medium, as seen from Table 6, there is no significant difference in the kinetic parameters of phenylalanine uptake between strains Y294 and Y294Δagp1. These results indicate that the absence of the Agp1p has little or no influence on phenylalanine transport in the presence of the high-affinity permease with *K*_*T*1_ = 11 *μ*M.

However, in MP-grown cells the disruption of *BAP2* (Y294Δbap2 strain) causes a substantial change in the apparent affinity constant for high-affinity phenylalanine uptake (*K*_*T*1_) from 11 to 4.7. The results with the Y294Δbap2 strain show that although Gap1p is largely responsible for L-phenylalanine uptake, there is apparently some contribution of Bap2p.

Later we analyzed the kinetics of phenylalanine uptake by cells grown in MA medium. For the strains Y294 and Y294Δbap2, the Eadie-Hofstee plots are clearly biphasic, whereas for the strain Y294Δagp1 the plot is linear. These results confirm that phenylalanine uptake is mediated by multiple permeases with different affinities and capacities.

The disruption of *BAP2* gene specifically results in the absence of the high-affinity phenylalanine uptake component (see Table 6). The apparent *K*_*T*_ value of Bap2 p was found to be 24 *μ*M and the *J*_max⁡_ value was 0.86 *μ*mol/min per g. of dry weight. The phenylalanine uptake kinetics for the strain Y294Δbap2 indicates that there is also a small contribution to phenylalanine uptake by two more permeases in strain Y294 grown in repressing (MA) conditions (Table 6). One of these has relatively high affinity, *K*_*T*_ = 67 *μ*M, while the other has low affinity for phenylalanine (*K*_*T*2_ = 0.77 mM).

Apparently, when *AGP1* is disrupted, only a minor part of phenylalanine uptake is abolished in MA-grown cells. The uptake data for the Y294Δagp1 strain shows the loss of the low-affinity Agp1p permease (*K*_*T*_ = 0.77 mM and *J*_max⁡_ = 0.46 *μ*mol/min/g (dry weight) observed in the parental strain). Thus, the kinetics of phenylalanine uptake in Y294Δagp1 did not deviate significantly from the kinetics of a single transport system owing to the presence of two permeases of similar affinity: Bap2p and the unknown phenylalanine permease (*K*_*T*_ = 67 *μ*M and *J*_max⁡_ = 0.21 *μ*mol/min/g of dry weight). This activity is not due to residual Gap1p activity under our experimental conditions (Table 2).

To further characterize the specificity of the unknown permease (*K*_*T*_ = 67 *μ*M) we measured uptake of 20 *μ*M L-[^14^C]- phenylalanine uptake in the presence of various amino acids at 200 *μ*M of concentration in Y294Δbap2 strain grown in MGln medium. Valine, tryptophan, tyrosine, isoleucine, and leucine inhibited the phenylalanine transport 30 to 50%.

Regenberg et al. [14] showed that in the strain *ssy1gap1* the overexpression of the *BAP3* gene, but not the *TAT2* gene (under control of the constitutive TPI1 promoter), caused an increase in the transport of branched-chain amino acids. The Bap3p and Tat2p permeases are also capable of transporting phenylalanine, tyrosine, and tryptophan. These results suggest that *BAP3* may encode the other unidentified phenylalanine permease with a km of 67 *μ*M.

Since Agp1p shows a high level of low-affinity phenylalanine uptake activity in strain Y294Δgap1 (Figure 1(d)), the kinetics of this permease was studied in both MP and MA media comparing Y294Δgap1 and Y294Δagp1Δgap1 strains (Table 6). In MP medium the removal of Agp1p activity led to the loss of the major part of the uptake capability and the residual uptake was linear with substrate concentration. The apparent *K*_*T*_ value of Agp1p was found to be 0.75 mM and the *J*_max⁡_ value was 14.3 *μ*mol/min per g. of dry weight. With ammonium ion as nitrogen source, the removal of Agp1p activity (strain Y294Δagp1Δgap1) also affects the total phenylalanine uptake capability, but to a lesser degree. At least two components are involved in phenylalanine uptake in Y294Δagp1Δgap1 strain, as shown by the Eadie-Hofstee plot. The kinetic analysis indicates the presence of a nonsaturable component and one transport system of high-affinity with *K*_*T*_ of 39 *μ*M and *J*_max⁡_ of 0.60 *μ*mol/min per g. of dry weight. No significant difference was seen between the Y294Δbap2Δgap1 and Y294Δgap1 strains in either medium (data not shown).

## 4. Discussion

The experiments in this study indicate that the transport of L-phenylalanine by *Saccharomyces cerevisiae* is catalyzed primarily by three permeases, Gap1, Bap2p, and Agp1p. The relative contribution of these permeases depends upon the type of nitrogen source, the concentration of phenylalanine, and the presence or absence of disruption mutations in one or more of the genes encoding the three permeases. Uptake values for phenylalanine are measured at a pH of 4.5 units. We consider that this condition is similar to the pH value of the growth medium in which amino acids uptake is required for cell growth. This does not imply that the relative participation of the different transport systems on phenylalanine uptake could not vary according to changes in pH values.

Phenylalanine transport in wild type cells grown on medium containing proline as sole nitrogen source (MP medium) is catalyzed by Gap1p, as expected [11, 27]. When ammonium ion is used as nitrogen source, (MA medium) Gap1p activity is absent while phenylalanine uptake remains almost the same. The major permease responsible for high-affinity phenylalanine transport is Bap2p, rather than the broad-specificity permease, Agp1p (Figure 1). Disruption of *BAP2* results in a dramatic loss of phenylalanine transport activity, whereas disruption of *AGP1* has no effect. At relatively high phenylalanine concentration (1.0 mM) a significant fraction of phenylalanine transport involves two more permeases, one of these has intermediate affinity with an apparent *K*_*T*_ of 67 *μ*M (Table 6). The permease responsible for this activity has not been identified but is not Agp1p (Figure 1(b), Table 6). In addition, *GAP1* disruption in a bap2 background causes resistance to the phenylalanine analogue *β*-(2-thienyl) alanine.

Participation of Agp1p in phenylalanine transport is also demonstrated by the growth defect observed when cells are grown on minimal medium containing 1.0 mM phenylalanine as sole nitrogen source (data not shown). It may be noted that although GAP1 was functional in this experiment it was not the only permease involved in phenylalanine uptake. This result extends the experiments of Iraqui et al. [13] who found that disruption of both *GAP1* and *AGP1* abolished growth on a similar medium.

It is important to emphasize that in our experiments, except for those in Table 1, 0.23 mM leucine was present in all growth media. This ensured that transcription of both *AGP1* and *BAP2* would be fully activated by SPS-dependent signaling [21].

One of the surprising conclusions which can be drawn from our results is that inactivation/repression of *GAP1* permease is not equivalent to *GAP1 *disruption. The difference is particularly striking when the effects of *AGP1* disruption on phenylalanine transport in MA-grown wild type cells and in *agp1gap1* cells are compared. *AGP1* disruption alone caused no decrease in either high- or low-affinity phenylalanine uptake relative to wild type (Figures 1(a) and 1(b)). In contrast, *AGP1* disruption caused dramatic decreases in uptake in *gap1* cells (Figures 1(c) and 1(d)). The *GAP1* disruption in MP-grown cells increases low-affinity phenylalanine uptake more than eightfold (Figures 1(b) and 1(d)). Most (76%) of this high uptake is lost upon introduction of the *agp1* mutation into the genome. Similarly, 83% of high-affinity phenylalanine uptake can be attributed to Agp1p (Figure 1(c)). Apparently the presence of Gap1p protein in MP-grown wild type cells limits production of active Agp1p. Perhaps inactivated Gap1p plays a similar role in wild type cells grown in MA medium.

Y294Δgap1 cells grown in MA medium exhibit some loss of low-affinity phenylalanine transport in response to disruptions in either BAP2 or AGP1. These responses may be additive (Figure 1(d)). In this case both Agp1p and Bap2p are contributing to low-affinity phenylalanine transport simultaneously. The competitive inhibition studies detailed in Tables 3, 4, and 5 reveal some surprising dynamic relationships between the three principal permeases that can catalyze phenylalanine uptake. These experiments provide an alternative way of identifying which permeases are transporting phenylalanine in different mutants grown in different media. Table 3 shows that for cells grown in MP medium, disruption of *BAP2 *increases inhibition by citrulline, indicating a greater contribution by Gap1p in high-affinity phenylalanine uptake. As expected, trifluoroleucine inhibition is decreased in the *bap2* strain. Taken together, the results indicate that Bap2p does in fact contribute to high-affinity phenylalanine uptake in MP-grown cells.

As noted above (Figure 1(d)), disruption of *GAP1* causes a major change in the behavior of Agp1p, but little or no change in Bap2p (Table 5). Disruption of *AGP1* in MP-grown *gap1* cells almost eliminates sensitivity of phenylalanine uptake to the three amino acids tested, and uninhibited transport was severely limited. In contrast, disruption of *BAP2* had no appreciable effect.

Although the *agp1gap1* strain retains the *BAP2* gene, apparently little or no Bap2p is expressed in MP-grown cells, since competition between high-affinity phenylalanine uptake and leucine is barely detectable (Table 5). Moreover, high-affinity phenylalanine uptake in the absence of inhibitors is very low. In contrast, when the cells are grown in MA medium, high-affinity phenylalanine uptake more than doubles, and inhibition by leucine is restored. That reflects that *BAP2* expression is supported by the strong competition of trifluoroleucine and *β*-(2-thienyl) alanine.

The kinetic studies reveal another surprising effect; namely, at high phenylalanine concentration in MP-grown cells, a significant level of the phenylalanine transport appears to either be nonsaturable or have an extremely low affinity (Table 6). This component does not appear in MA-grown cells of wild type, or in mutants with single disruptions in *BAP2* or *AGP1* (Table 6). Here again, the disruption of *GAP1* has very different effects on phenylalanine transport systems than repression and inactivation of *GAP1* by ammonium ion. As we found in this work, there is an additional phenylalanine transporter, it remains for future studies to establish the nature of that system. A plausible approach would be to study the transport of phenylalanine in gap1, agp1, and bap2 triple mutants.

## Figures and Tables

**Table 1:** Phenylalanine transport in a Y294Δgap1 strain at different leucine concentrations.

L-leucine (*µ*M)	Initial velocity (nmol·g^−1^·min^−1^)
0	138 ± 6
10	142 ± 3
50	164 ± 6
230	227 ± 23

Cells were grown in MA medium plus all supplements plus leucine at the indicated concentrations.

L-[^14^C]-phenylalanine concentration was 20 µM.

**Table 2:** Initial velocities of L-citrulline uptake.

Strains	MP medium	MA medium
Y294	103 ± 4	<6
Y294Δbap2	301 ± 13	<6
Y294Δagp1	189 ± 8	<6
Y294Δgap1	<6	<6

Initial velocity expressed in nmol·g^−1^·min^−1^. L-[^14^C]-citrulline concentration was 20 *μ*M.

**Table 3:** Specificity of phenylalanine uptake in cells grown in MP^a^.

Unlabelled amino acid added	Percentage inhibition		
	Y294	Y294Δbap2	Y294Δagp1
Leucine	76	75	78
Isoleucine	71	71	73
Valine	52	71	56
Tyrosine	60	81	66
Tryptophan	48	86	60
Asparagine	18	37	19
Citrulline	15	40	20
DL-trifluoroleucine	59	42	62

^a^High-affinity phenylalanine uptake was measured at 4 min with L-[^14^C]-phenylalanine 20 *μ*M and the unlabelled amino acids were added at 200 *μ*M. Control values in the absence of competitor in strains Y294, Y294Δbap2, and Y294Δagp1 were 2.31, 1.75, and 2.76 *μ*mol g^−1^, respectively.

**Table 4:** Specificity of phenylalanine uptake in cells grown in MA medium^a^.

Unlabelled amino acid added	Percentage inhibition		
	Y294	Y294Δbap2	Y294Δagp1
Leucine	76	34	72
Proline	6	0	0
DL-TFL^b^	57	3	48
*β*-2TA^c^	71	26	67

^a^High-affinity phenylalanine uptake was measured at 4 min with L-[^14^C]-phenylalanine 20 *μ*M and the unlabelled amino acids were added at 200 *μ*M. Control values in the absence of competitor in strains Y294, Y294Δbap2, and Y294Δagp1 were 2.29, 0.32, and 1.91 *μ*mol g^−1^, respectively.

^b^DL-trifluoroleucine.

^c^ *β*-2-thienylalanine.

**Table 5:** Specificity of phenylalanine uptake in gap1 cells grown in MP and MA media^a^.

Unlabelled amino acid added	Percentage inhibition					
	Y294Δgap1		Y294Δbap2Δgap1		Y294Δagp1Δgap1	
	MP	MA	MP	MA	MP	MA
Leucine	39	45	34	34	4	51
Tryptophan	17	18	18	25	4	23
Asparagine	45	29	48	35	4	0
Proline	ND	8	ND	13	ND	12
DL-TFL^b^	ND	30	ND	3	ND	37
*β*-2TA^c^	ND	43	ND	31	ND	55

^a^High-affinity phenylalanine uptake was measured at 4 min with L-[^14^C]-phenylalanine 20 *μ*M and the unlabelled amino acids were added at 200 *μ*M. Control values in the absence of competitor in strains Y294Δgap1, Y294Δbap2Δgap1, and Y294Δagp1Δgap1 grown in MP medium were 1.89, 1.63, and 0.44 and for cells grown in MA medium were 1.59, 0.98, and 1.09 *μ*mol g^−1^, respectively.

^b^DL-trifluoroleucine.

^c^ *β*-2-thienylalanine.

**Table 6:** Kinetic parameters of L-phenylalanine transport.

Strains	Medium	*K*_*T*1_ (*μ*M)	*J*_max⁡1_	*K*_*T*2_ (mM)	*J*_max⁡2_	NS
Y294	MP	11 ± 2	0.85 ± 0.02	0.77 ± 0.3	0.46 ± 0.05	0.42 ± 0.02
	MA	24 ± 5 67 ± 26	0.86 ± 0.08 0.21 ± 0.07			—

Y294Δbap2	MP	4.7 ± 1	0.58 ± 0.03	—	—	0.46 ± 0.02
	MA	67 ± 26	0.21 ± 0.07	0.77 ± 0.3	0.46 ± 0.05	—

Y294Δagp1	MP	11 ± 1	1.04 ± 0.04	—	—	0.47 ± 0.03
	MA	26 ± 2	1.37 ± 0.02	—	—	—

Y294Δgap1	MP	—	—	0.75 ± 0.3	14.3 ± 2.0	2.97 ± 1.10
	MA	39	0.34	0.70	5.09	2.66

Y294Δagp1Δgap1	MP	—	—	—	—	2.91
	MA	39	0.6	—	—	2.55

L-[^14^C]-phenylalanine uptake was assayed as described in Section 2. Uptake data were plotted as Michaelis-Menten and Lineweaver-Burk plots (data not shown) and the kinetic parameters estimated using the Solver program of Microsoft Excel.

NS: nonspecific (*K*_*T*_ ≥ 1 M).
